# Molecular epidemiology and characteristic of virulence gene of community-acquired and hospital-acquired methicillin-resistant *Staphylococcus aureus* isolates in Sun Yat-sen Memorial hospital, Guangzhou, Southern China

**DOI:** 10.1186/s12879-016-1684-y

**Published:** 2016-07-22

**Authors:** Xiaoying Xie, Yunwen Bao, Nengyong Ouyang, Xinlu Dai, Kunyi Pan, Baiji Chen, Yawen Deng, Xiquan Wu, Fengqin Xu, Hongyu Li, Songyin Huang

**Affiliations:** Department of Laboratory, Guangdong Provincial Key Laboratory of Malignant Tumor Epigenetics and Gene Regulation, Sun Yat-Sen Memorial Hospital, Sun Yat-Sen University, Guangzhou, 510120 China; Department of Gynaecology and Obstetrics, Sun Yat-Sen Memorial Hospital, Sun Yat-Sen University, Guangzhou, 510120 China

**Keywords:** Methicillin-resistant *Staphylococcus aureus*, Antimicrobial susceptibility, Molecular characteristics, Virulence gene

## Abstract

**Background:**

Methicillin-resistant *Staphylococcus aureus* (MRSA) is a major cause of both hospital and community infections globally. It’s important to illuminate the differences between community-acquired MRSA (CA-MRSA) and hospital-acquired MRSA (HA-MRSA), but there have been confusions on the definition, especially for the MRSA isolates identified within 48 h of admission. This study aimed to determine the molecular characteristics and virulence genes profile of CA and HA-MRSA isolates identified less than 48 h after hospital admission in our region.

**Methods:**

A total 62 MRSA isolates identified within 48 h after admission and the clinical data were collected. Antimicrobial susceptibility test (AST) of collected isolates were performed according to the guidelines of Clinical and Laboratory Standards Institute (CLSI) 2015, and staphylococcal cassette chromosome mec (SCC*mec*) typing, multilocus sequence typing (MLST), pulsed-field gel electrophoresis (PFGE) and virulence gene profiling were performed to explore the molecular diversity.

**Results:**

SCC*mec* III and sequence type (ST) 239 were the most prevalent SCC*mec* type and ST in both CA and HA-MRSA groups. HA-MRSA group had higher prevalence of SCC*mec* III (87.2 %) and ST239 (79.5 %) compared with CA-MRSA (60.9 and 43.4 %, both *P* < 0.001), while the frequency of SCC*mec* IV (26.0 %) and ST59 (21.7 %) were higher in CA-MRSA than its counterpart (*P* < 0.001 and *P* = 0.003). MRSA-ST239-III was the predominant type in this study (61.3 %, 38/62), especially in HA-MRSA group (76.9 %, 30/39). However, CA-MRSA strains exhibited more diversity in genotypes in this study. Meanwhile, CA-MRSA tended to have lower resistant percentage to non-β-lactams antibiotics but more virulence genes carriage, especially the staphylococcal enterotoxins (SE) genes. Notably, *seb* gene was only detected in CA-MRSA isolates (52.2 %), likely a significant marker for CA-MRSA isolates. Panton-Valentine leukocidin gene (PVL) was highly detected in both groups, while appeared no significantly different between CA-MRSA (47.8 %) and HA-MRSA (43.6 %).

**Conclusions:**

Our findings support a difference in the molecular epidemiology and virulence genes profile of CA-MRSA and HA-MRSA. Furthermore, this study indicates a possible transmission from HA-MRSA to CA-MRSA, which may cause the overlap of the definition.

**Electronic supplementary material:**

The online version of this article (doi:10.1186/s12879-016-1684-y) contains supplementary material, which is available to authorized users.

## Background

Methicillin-resistant *Staphylococcus aureus* (MRSA) is a major cause of nosocomial infections around the world since 1960s, first reported in England [1], and the increasing prevalence rates were noticed by the researchers in recent years [2, 3]. Otherwise, the epidemiology of MRSA infection has changed in the past decades because of the emergence of the strains acquired outsides the healthcare environment named community-associated *Staphylococcus aureus* (CA-MRSA) [4]. According to the previous reports, CA-MRSA isolates have somewhat differences from hospital-acquired MRSA (HA-MRSA) isolates in molecular characterization, antibiotic resistant pattern, pathogenicity and virulence factors [5].

As reported, the predominant HA-MRSA clones in China are MRSA-ST239-III and MRSA-ST5-II [6]. In contrast, the main CA-MRSA clones are MRSA-ST59-VI/V and its single locus varietas MRSA-ST338-IV/V. It was suggested that most CA-MRSA isolates carry Staphyloccoccal cassette chromosome mec (SCC*mec*) types IV and V, do not have multi-antibiotic resistance (except to β lactams) [7, 8]. However, in some studies recently, SCC*mec* IV was identified in HA-MRSA isolates and SCC*mec* III was identified in both HA-MRSA and CA-MRSA isolates, showed that the two SCC*mec* typing isolates may spread between hospital and community in some areas [9]. The overlap of molecular characteristic of CA-MRSA and HA-MRSA was eminently possible in the MRSA isolates which identified within 48 h after admission to hospital [10, 11]. As the main metropolises with a large population of residents and visitors in Southern China, Guangzhou may have an unique transmission pattern of HA-MRSA and CA-MRSA, especially the MRSA strains obtained within 48 h of admission, while rarely studied.

Furthermore, CA-MRSA isolates posses different virulence gene profiles to their counterparts, such as genes, coding a leukocidin well known for its apoptosis induction in human polymorphic leukocytes and its lytic effects in the cells. Since the confusion about definitions of CA-MRSA and HA-MRSA isolates by traditional method basic on clinical and epidemiological criterion, the specific virulence gene (PVL or *LukF/lukS-PV*) become a new molecular marker [11, 12]. Besides, CA-MRSA was also shown to have higher expression levels of hemolysins and superantigens, such as staphylococcal enterotoxins gene *sek* and *seq*, suggesting that they are more virulent than HA-MRSA [11]. It can be supposed that these virulence genes may be new candidates of classified markers, which were few reported as well.

In order to understand the molecular epidemiology and characterization of virulence gene of CA-MRSA and HA-MRSA isolates in this region, 39 HA-MRSA and 23 CA-MRSA isolates were collected from Sun Yat-Sen Memorial Hospital from Jan.1, 2006 to Dec.31, 2011. These isolates were genotyped by SCC*mec* typing and multilocus sequence typing (MLST). The genetic relationship of the isolates was determined by pulsed-field gel electrophoresis (PFGE). The presence of virulence genes in CA-MRSA and HA-MRSA isolates were determined to establish their possible relationship to clinical outcomes. This knowledge will contribute to our understanding of how the MRSA epidemic has evolved.

## Methods

### Study design and population

Sun Yat-Sen Memorial Hospital is also known as the Second Affiliated Hospital of Sun Yat-Sen University. With more than 4200 staff and 2200 inpatient beds available, the hospital performs over 50,000 inpatient operations, discharges about 80,000 inpatients, and handles more than 3 million outpatient visits annually. A hospital-based retrospect study of 587 inpatients hospitalized for < 48 h infected by *Staphylococcus aureus*, included 67 inpatients with MRSA infection in Sun Yat-Sen Memorial Hospital between Jan.1, 2006 and Dec.31, 2011 were carried out. Five patients with MRSA infection were eliminated because the strains can’t recovery. Therefore, a total of 62 compete surveys were obtained, with an efficiency rate of 92.5 %. Based on the US Centers for Disease Control and Prevention criteria, we roughly defined HA-MRSA infections as those patients who fit any one of the following situations: surgery, haemodialysis, residence in a long-term care facility or treatment in hospital during the previous year, presence of a permanent catheter or percutaneous device at the time of culture, or previous isolation of MRSA. If the patients did not meet any of the above risk factors, had an infection at the time of admission, and the culture of the infection was taken within 48 h, then the infection was considered as CA-MRSA infections. Clinical data related to the type of infection and outcome (death, improvement, and relapse) of the patients, as well as other data, were collected for up to a period of 30 days after the diagnosis of infection by MRSA. All patients, parents or guardians signed informed consent approving the use of their specimen samples for research purposes and the study was approved by the Ethics Committee of Sun Yat-Sen Memorial Hospital. Ethical committee’s reference number: [2016]伦备第 (11)号 (Additional file 1).

### Bacterial strains

According to the above definition, 39 HA-MRSA isolates and 23 CA-MRSA isolates were collected from the 62 patients. To avoid sample duplication, isolates that were consecutively isolated from the same individual were excluded. All isolates underwent phenotypic identification using the VITEK® 2 microbial identification system (bioMérieux, Marcy l’Etoile, France) according to the manufacturer’s instructions. Antibiotics used for susceptibility testing included penicillin, erythromycin, clindamycin, cefuroxime, ceftriaxone, cefoxitin, gentamicin, rifampicin, quinupristin/dalfopristin, trimethoprim/sulfamethoxazole, cefotaxime, tetracycline, imipenem, teicoplanin, vancomycin, ciprofloxacin, and moxifloxacin. Susceptibilities were determined using the disk diffusion method in accordance with the performance standards for antimicrobial susceptibility testing, 25^rd^ informational supplement (M100-S25), recommended by the Clinical and Laboratory Standards Institute. The inducible clindamycin resistance was determined by D-test. All disks were obtained from Oxoid Ltd (Oxoid, Basingstoke, England), and *S. aureus* ATCC 25923 and ATCC29213 were used as the quality control strain.

### Molecular characterization

Bacterial DNA was extracted using DNA extracted kit(Tiangen Biotech, Beijing) with lysostaphin according to the manufacturer’s instructions. The sequence type (ST) was characterized by MLST, and the products of seven house-keeping gene fragments were sequenced (Sangon Biotech, Shanghai) and compared with allele profiles from database of *S. aureus* (www.mlst.net/). SCC*mec* typing of MRSA strains was conducted by multiplex PCR method as previously described [13]. The genetic relationship of the isolates was determined by PFGE as previously described [18]. Cluster analysis was performed with the software program BioNumerics 5.0 (Maths, Belgium) using the Dice coefficient and the unweighted pair group method (UPGMA). The isolates with the similarity over 75 % were clustered in patterns. The presence of the genes that code for PVL (*lukF-PV* and *lukS-PV*), twelve staphylococcal enterotoxins (*sea ~ see, seg ~ sej* and *sem-seo*), two exfoliative toxins (*eta* and *etb*), four hemolysins (*α-hemolysin*, *β-hemolysin*, *δ-hemolysin* and *γ-hemolysin*), and the toxic shock syndrome toxin (*tsst*-1) and *mecA* gene was determined using PCR as previously reported methodologies, with some modifications [14, 15].

### Statistical analysis

In descriptive statistics, frequency and proportions were calculated for categorical variables. The frequency of SCC*mec* type, specimen type and virulence genes were treated as categorical variables. The chi-square or two-sided Fisher’s exact test was used to discriminate whether the distributions of the studied genes or types were significantly different between HA-MRSA and CA-MRSA. The only continuous variable, age, was transformed into a categorical variable using the quartiles of the frequency distribution (~18, 19–60, >60). It was considered statistically significant if the two-side *P*-value < 0.05. All statistical analyses were carried out using SPSS 19.0 for Windows (IBM). All susceptibility data and molecular test results were analyzed using WHONET software, version 5.6.

## Result

### Clinical and epidemiological data

A total of 587 inpatients hospitalized for < 48 h infected by *Staphylococcus aureus*, included 67 inpatients with MRSA infection between Jan.1, 2006 and Dec.31, 2011. Five patients with MRSA infection were eliminated because the strains can’t recovery. Therefore, a total of 62 compete surveys with 62 MRSA isolates were obtained. Thirty-nine isolates (62.9 %) were classified as HA-MRSA and 23 isolates (37.1 %) were classified as CA-MRSA. HA-MRSA strains were mainly isolated from patients over 60 years, while CA-MRSA strains were from the younger ones. The CA-MRSA strains were generally isolated from skin lesion secretion and pus (totally 86.9 %, 20/23), causing infection of skin and soft tissue. HA-MRSA strains were isolated mainly from sputa (56.4 %, 22/39), causing pneumonia. Totally 61.3 % of the patients infected by MRSA had other chronic disease ever before, like diabetes, hypertension, tumor and autoimmune disorders. Furthermore, HA-MRSA isolates preferred infecting the patients suffered from tumor and brain disease as brain trauma and cere-brovascular disease (totally 35.9 %), while had a better outcome than the CA-MRSA strains. Spring and autumn were the most popular seasons for CA-MRSA isolates, while HA-MRSA infection often occurred in summer and autumn (Details in Table 1).

**Table 1:** Clinical features of the HA-MRSA and CA-MRSA strains

Characteristic	CA-MRSA	HA-MRSA	*P*-value
	(*n* = 23)	(*n* = 39)	
	n(%)	n(%)	
Sex			
Male	11(47.8)	24(61.5)	0.427
Female	12(52.2)	15(38.5)	
Age			
~ 18	5(21.7)	3(7.7)	0.149
19-60	11(47.8)	16(41.0)	
> 60	7(30.4)	20(51.3)	
Season			
Mar. ~May	9(39.1)	1(2.6)	0.001
Jul. ~Aug.	2(8.7)	14(35.9)	
Sep. ~Nov.	7(30.4)	15(38.5)	
Dec. ~Feb.	5(21.7)	9(23.1)	
Clinical source			
Skin lesion	15(65.2)	16(41.0)	0.001
Pus	5(21.7)	1(2.6)	
Sputa	2(8.7)	22(56.4)	
Bone	1(4.3)	0(0)	
Type of infection			
Infection of skin and soft tissue	20(87.0)	11(28.2)	<0.001
Pneumonia	2(8.7)	22(56.4)	
Infection of the surgical site	0(0)	6(15.4)	
Infection of bone and joint	1(4.3)	0(0)	
Underline disease			
None	11(47.8)	13(33.3)	0.147
Diabetes	5(21.7)	5(12.8)	
Hypertension	6(26.1)	9(23.1)	
Tumor	0(0)	4(10.3)	
Brain injury	1(4.3)	10(25.6)	
Other	2(8.6)	6(15.4)	
Outcomes			
Relapse	5(21.7)	9(23.1)	0.809
Improvement	17(73.9)	25(64.1)	
Death within 1 month	1(49.4)	3(7.7)	

### Antimicrobial susceptibility

**Fig. 1:**
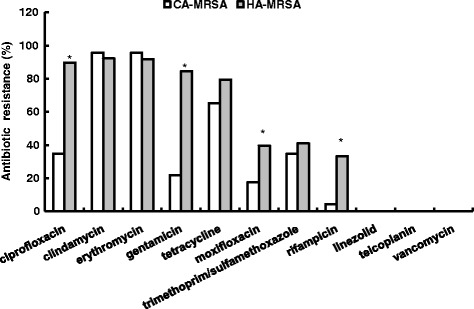
Antibiotic resistance rates of 62 MRSA isolates. CA-MRSA vs. HA-MRSA, *P < 0.05

All isolates were susceptible to vancomycin, teicoplanin and linezolid. HA-MRSA strains had higher resistant percentage than CA-MRSA to the non-β-lactams antibiotics, including ciprofloxacin (89.7 % vs. 34.8 %, *P* < 0.001), moxifloxacin (39.5 % vs. 17.4 %,*P* = 0.001), rifampicin (33.3 % vs. 4.3 %,*P* < 0.001), and gentamicin (84.6 % vs. 21.7 %,*P* < 0.001). High resistance rate to erythromycin (95.7 and 91.9 %) and clindamycin (95.7 and 92.3 %) were detected from both the CA-MRSA and HA-MRSA isolates, followed by tetracycline (65.2 and 79.4 % respectively) (Fig. 1 and Additional file 2: Table S1).

### Molecular characteristics of HA-MRSA and CA-MRSA

**Fig. 2:**
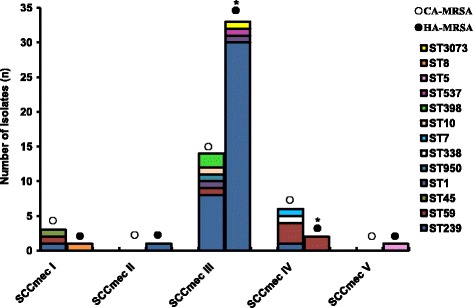
SCC*mec* type and ST distribution of the 62 MRSA isolates. CA-MRSA vs. HA-MRSA, *P < 0.05. CA-MRSA, community-acquired methicillin-resistant *Staphylococcus aureus*; HA-MRSA, hospital-acquired methicillin-resistant *Staphylococcus aureus*; SCC*mec*, staphylococcal cassette chromosome mec; ST, sequence types

The distribution of SCC*mec* types in the 62 MRSA strains is shown in Fig. 2 and Additional file 2: Table S2. Among these, SCC*mec* type III was found in 48 isolates (77.4 %), followed by type IV (12.9 %) and type I (6.5 %) of the isolates, respectively. It is interesting to note that the prevalence of the SCC*mec* type III was significantly higher in isolates from HA-MRSA compared with those from CA-MRSA (87.2 % vs. 60.9 %, *P* < 0.001), whereas the SCC*mec* type IV was more common among isolates from CA-MRSA (26.0 %. vs. 5.1 %) (*P* < 0.001). In addition, SCC*mec* II and SCC*mec* V clones were only found in the HA-MRSA isolates.

Multilocus sequence typing of 62 MRSA strains revealed 12 different sequence types. Among these, ST239 and ST59 the most prevalent, accounting for 66.1 and 11.3 % of all isolates, respectively. The prevalence of the ST239 was significantly higher in isolates from HA-MRSA compared with those from CA-MRSA (79.5 % vs. 43.4 %, *P* < 0.001), whereas the ST59 was more common among isolates from CA-MRSA (21.7 % vs. 5.1 %, *P* = 0.003). ST45, ST950, ST338, ST7, ST10 and ST398 were only found in the CA-MRSA group; ST537, ST5 and ST8 were found in HA-MRSA group only. Additionally, a new ST clone ST3073 (3-1-1-8-345-1-1) (ID:5611) was found in the HA-MRSA group, obtained from nasal secretion of a 26 years female patients in 2009, who suffered from a nasopharyngeal surgical site infection. Besides, one HA-MRSA was untypable (Additional file 2: Table S3). In short, MRSA-ST239-III were the most prevalent of all isolates (61.3 %, 38/62), which was found in 30 (76.9 %) HA-MRSA isolates and eight (34.8 %) CA-MRSA isolates (Fig. 2 and Additional file 2: Table S4).

### Virulence factors

The results of virulence gene analysis are shown in Table 2. Almost all the isolates contained *hla* (100 %, 62/62), *hlb* (90.3 %, 56/62) and *hlg* (98.4 %, 61/62) genes but no isolate was *hld* positive. The *sea* gene was found in 37 isolates (59.7 %) and 13 isolates (21.0 %) possessed the *sei* gene. The *seb* and *sec* gene were found in 12 isolates (19.4 %). The *sem* was found in seven isolates (11.3 %). Six isolates (9.7 %) possessed the *seo* gene and five isolates (8.1 %) possessed the *sen* gene. The *sed, see* and *sej* genes were not found. These 12 genes make up the enterotoxin gene cluster (egc). The *tsst-1* gene was found in three isolates (4.8 %), exfoliative toxins genes (*eta* or *etb*) were not found. The PVL gene were found in 11 CA-MRSA isolates (47.8 %) and 17 HA-MRSA isolates (43.6 %) and there were no significant differences between CA and HA-MRSA strains (Table 2, Additional file 3: Figure S1).

**Table 2:** Detection of the 20 virulence genes of 62 MRSA isolates in this study

Virulence genes	No. Of positive isolates	Virulence genes carriage of *S. aureus*, n (%)		*P*-value
		CA-MRSA	HA-MRSA	
	(*n* = 62)	(*n* = 23)	(*n* = 39)	
*sea*	37(59.7)	13(56.5)	24(61.5)	0.284
*seb*	12(19.4)	12(52.2)	-	
*sec*	12(19.4)	7(30.4)	5(12.8)	0.039
*seg*	12(19.4)	3(13.0)	9(23.1)	0.097
*seh*	2(3.2)	-	2(5.1)	
*sei*	13(21.0)	4(17.4)	9(23.1)	0.377
*sem*	7(11.3)	4(17.4)	3(7.7)	0.086
*sen*	5(8.1)	3(13.0)	2(5.2)	0.081
*seo*	6(9.7)	4(17.4)	2(5.1)	0.011
*hla*	62(100.0)	23(100.0)	39(100.0)	
*hlb*	56(90.3)	20(87.0)	36(92.3)	0.357
*hlg*	61(98.4)	23(100.0)	38(97.4)	0.545
*tsst*-1	3(4.8)	2(8.7)	1(2.6)	0.134
PVL	28(45.2)	11(47.8)	17(43.6)	0.670

*Sea ~ seo,* gene encoding staphylococcal enterotoxins; *hla ~ hlg*, gene encodingα-hemolysin ~ γ-hemolysin; *tsst*-1, gene encoding toxic shock syndrome toxin 1; PVL*, g*ene encoding Panton-Valentine leukocidin

Among isolates classified as CA-MRSA, the detection rates of *sec* and *seo* were higher than HA-MRSA group (*P* < 0.05) and all the *seb* positive isolates were CA-MRSA strains (52.2 %, 12/23). On the other hand, *seh* gene was only found in HA-MRSA isolates (5.1 %, 2/39). Furthermore, 65.2 % CA-MRSA strains carried over than five virulence genes, were obviously higher than that in HA-MRSA group (35.9 %, *P* < 0.05).

Table 3 shows the association between clinical diagnosis and the presence of virulence genes. The *sea*, *hla*, *hlb*, and *hlg* genes were detected in a higher proportion in patients with MRSA infection. There are no significantly different between the different types of infection groups. The *sea* and PVL genes were detected in a higher proportion in patients with pneumonia. But the *sem*, *sen*, *seo*, *tsst*-1 genes were not found in MRSA isolates causing pneumonia. Of note, the enterotoxin gene cluster (*sea*, *seg*, *sei*, *sem*, *sen and seo*) was detected in a higher proportion in patients with surgical site infection by HA-MRSA (*P* < 0.001). However, the *seb* and *sec* genes were significantly associated with skin and soft tissue infection (*P* < 0.001), and proved to be significant markers of CA-MRSA isolates.

**Table 3:** Detection of the virulence genes of MRSA in accordance with the clinical diagnosis of the associated infection

Virulence gene	Number and percentage of the MRSA isolates, n(%)			*P*-value
	Infection of the surgical site	Infection of skin and soft tissue	Pneumonia	
	(*n* = 6)	(*n* = 31)	(*n* = 24)	
*sea*	4(66.7)	18(58.1)	15(62.5)	0.422
*seb*	-	11(35.5)	1(4.2)	<0.001
*sec*	1(16.7)	6(19.4)	4(16.7)	<0.001
*seg*	4(66.7)	4(12.3)	3(8.9)	<0.001
*seh*	-	1(3.2)	1(4.2)	1.000
*sei*	3(50.0)	6(19.4)	3(8.9)	<0.001
*sem*	2(33.3)	4(12.3)	-	<0.001
*sen*	2(33.3)	2(6.6)	-	<0.001
*seo*	2(33.3)	3(9.7)	-	<0.001
*hla*	6(100.0)	31(100.0)	23(95.8)	
*hlb*	6(100.0)	27(87.1)	23(95.8)	<0.001
*hlg*	6(100.0)	30(96.8)	23(95.8)	0.235
*tsst*-1	-	3(9.7)	-	
PVL	3(50.0)	12(38.7)	12(50.0)	0.210

*Sea ~ see, seg ~ sej, sem ~ seo*, gene encoding staphylococcal enterotoxins; *eta* and *etb*, gene encoding exfoliative toxin A and B; *hla ~ hlg*, gene encoding α-hemolysin ~ γ-hemolysin; *tsst*-1, gene encoding toxic shock syndrome toxin 1; PVL*, g*ene encoding Panton-Valentine leukocidin

The infection of bone and joint group (*n* = 1) was not included in statistical analysis

### Genetic relationship by PFGE

Among the 62 *S. aureus* isolates, PFGE grouped 60 isolates into eight pulsotypes, while two isolates were untypable. Pattern A was the main PFGE cluster in this study (55.0 %, 33/60), followed by pattern B (20.0 %, 12/60). HA-MRSA strains mainly belonged to pattern A (66.7 %, 26/39) and pattern B (20.5 %, 8/39), while CA-MRSA didn’t show a predominant pattern as HA-MRSA strains (Table 4 and Additional file 4: Figure S2).

**Table 4:** The relationship of molecular characterization, antimicrobial resistance, and the main virulence factor profiles of 62 MRSA isolates

PFGE profile	SCC*mec*(n)	ST (n)	CA-MRSA (n)	HA-MRSA (n)	The main virulence factor profiles	Resistance profile
A(33)	I(2)	ST8(1)	0	1	*sea,* PVL*, hla,hlb,hlg*	CIP/CN/E/TET
		ST239(1)	0	1		
	III(30)	ST239(29)	7	22		
		ST1 (1)	0	1		
	V(1)	ST5(1)	0	1		
B(12)	III (11)	ST239(9)	2	7	*sea,seg, hla,hlb,hlg*	CIP/CN/E/TET/RD
		ST537(1)	0	1		
		ST1(1)	1	0		
	IVa(1)	7(1)	1	0		
C (8)	I(1)	ST59(1)	1	0	*sea,seb,seg,*PVL*, hla,hlb,hlg*	E/TET
	III(1)	ST59(1)	1	0		
	IVa(3)	ST59(3)	2	1		
	IVd(3)	ST59(1)	3	0		
		ST239(1)				
		ST338(1)				
D (2)	III(1)	ST950(1)	1	0	*seg,sei,sem ~ o,*PVL*, hla,hlb,hlg*	E
	II(1)	ST239(1)	0	1		
E (2)	III(2)	ST10(1)	1	0	*sea(seb),sec,seg(sei),sem ~ o,*PVL*,hla,hlb,hlg*	E
		ST3073(1)	0	1		
F (1)	I(1)	ST45(1)	1	0	*sec,seg,sei,*PVL*,hla,hlb,hlg*	E/TET
G (1)	III(1)	NT(1)	0	1	*hla,hlb,hlg*	E/TET/RD
H (1)	IVa(1)	ST59(1)	1	0	*seb,*PVL*, hla,hlb,hlg*	E/TET

*PFGE* pulsed-field gel electrophoresis, *SCCmec* staphylococcal cassette chromosome mec, *ST* sequence type, *Sea ~ see, seg ~ sej, sem ~ seo,* gene encoding staphylococcal enterotoxins; *eta* and *etb,* gene encoding exfoliative toxin A and B; *hla ~ hlg*, gene encodingα-hemolysin ~ γ-hemolysin; *tsst*-1, gene encoding toxic shock syndrome toxin 1; *PVL* Panton-Valentine leukocidin, *CIP* ciprofloxacin, *CN* gentamicin, *E* erythromycin, *TET* tetracycline, *RD* rifampicin, *NT* non-typable

The combinative analysis of electrophoretic pattern, SCC*mec* type, sequence type, virulence factor profile and drug resistance pattern suggested different resistance patterns and virulence factor profiles in different PFGE clusters. Combined with the isolates distribution, it indicated that CA-MRSA exhibited great diversity in genetic characteristic and tended to be less resistant to non-β-lactams antibiotics but more virulence genes carried. As the Table 4 showed, the isolates with pattern C, D, E and F, mainly CA-MRSA strains, often were resistant to one or two non-β-lactams antibiotics (TET/E), carried over than three SE genes besides the three hemolysin genes. In contrast, 87.9 % of the isolates with pattern A were MRSA-ST239-III, classified as HA-MRSA, often presented co-resistance to four or more non-β-lactams antibiotics (CIP/CN/E/TET), while only carried one or two SE genes besides the hemolysin genes (Table 4).

The eight PFGE gene clusters further divided into 22 genotypes. Due to the long time span of and different tissue resource of isolates, it indicated that there were not epidemic trend of MRSA strains between 2006 and 2011 in Guangzhou (Additional file 4: Figure S2).

## Discussion

MRSA is an increasing problem and its burden continues to rise in hospital and community setting, leading to increased morbidity and mortality rates. Despite the high prevalence, only a few epidemic clones have been identified in China. As reported, Brazilian or Hungarian clone (ST239) and the New York/Japan clone (ST5) were prevalent in some Asia countries, including China [6]. Previous studies from China found the majority HA-MRSA strains causing bloodstream infection in Shanghai belonged to above two epidemic clones ST239 and ST5 [16]. Wang et al. reported the first molecular characterization of CA-MRSA in 2004, belonged to ST59 [17]. Since then, molecular epidemiology of CA-MRSA in China had aroused more and more concern. During the last decade, ST59 (and its single locus variant ST338) was the major lineage to the CA-MRSA isolates in China [8, 14]. Consistently in this study, ST239 was the predominant clone among HA-MRSA strains, whereas, it is also detected among CA-MRSA. The result showed that 76.9 % (30/39) ST239 MRSA isolates belonged to PFGE cluster A suggesting a similar epidemiology for both hospital acquired infections and community acquired infections.

According to the previous reports [18], HA-MRSA is usually detected with SCC*mec* type I, II, and III; in contrast, CA-MRSA is reported as carrying type IV and V. In this study, SCC*mec* III was the dominant type in HA-MRSA strains (87.3 %), also the main type of CA-MRSA (60.9 %). This study confirms that CA-MRSA clones partially carry the SCC*mec* type III and SCC*mec* type I, as reported in a recent study in Guangzhou [16]. In previous studies from the USA [5] and Italy [10], SCC*mec* type IV were also found in HA-MRSA. This finding demonstrates the existence of constant genetic change in not just CA-MRSA but also in HA-MRSA. These bacteria undergo genetic variations that can give rise to isolates with greater capacities for environmental adaption.

It’s widely assumed that there are two sources of CA-MRSA: the spread of HA-MRSA from the medical staff and discharged patients, and the community MSSA strains acquired the resistance gene *mecA*. MRSA strains colonized in the nasal vestibule of hospital staff and patients can lead the spread in ward [3]. Our recent study also showed that hospital workers had a high risk of MRSA nasal carriage, and the carriers may ‘impose’ their carrier status upon other household members, causing transmission of HA-MRSA strains to the community [19]. In this study, the CA-MRSA-ST239-III strains had a high homology with HA-MRSA, shared the same PFGE cluster (Patterns A and B), similar virulence factor profiles and resistance patterns, indicated the strains’ transmission between healthy-care set and community can lead overlap in the distinction between HA-MRSA and CA-MRSA.

MRSA strains emerged and diffused in the community (CA-MRSA) and were shown frequently to carry the smaller and more easily mobilized SCC*mec* cassettes and are generally less drug resistant than their counterparts acquired in hospitals (HA-MRSA) [11]. Unexpectedly, except for resistance to all kinds of β-lactam antibiotics, the MRSA isolates found in our region appeared a high resistant rate to other non-β-lactam antibiotics, especially to erythromycin (93.3 %), clindamycin (93.5 %) and tetracycline (74.2 %), mainly in HA-MRSA strains of ST239-III in Pattern A. Unlike the HA-MRSA strains, CA-MRSA strains exhibited great diversity in genetic characteristic in this study. Except for the MRSA-ST239 and ST59, another seven unique sequence type constituted almost half of 23 CA-MRSA strains, belonged to Pattern C ~ H respectively. In comparison with HA-MRSA, these CA-MRSA isolates showed high levels of sensitivity to ciprofloxacin, moxifloxacin, gentamicin, rifampicin, and trimethoprim/sulfamethoxazole. These results are consistent with previous studies [20–22].

To our knowledge, this is the first study to provide insight into the epidemiological and genetic correlation of the virulence profile present among the different CA-MRSA and HA-MRSA clones and their clinical diagnosis in Mainland China. Hemolysin of *S. aureus* leads the bacterio-lytic enzyme release to damage the nearby organizations through effecting the red blood cell, platelet and neutrophil [23]. α-hemolysin and β-hemolysin are the main toxin inducing pathological injury. Consistent with the report in China [23], almost all the isolates contained *hla*, *hlb* and *hlg* gene but no isolate was *hld* positive. Difference from our report, the *hla*, *hlb*, *hlg* and *hld* were all found in Wenzhou [24]. These findings indicated that the high *hla*, *hlb* and *hlg* genes but low *hld* gene present in the MRSA strains could be a toxin characteristics of MRSA strains in this region.

In terms of virulence factors, differential content was reported between CA-MRSA and HA-MRSA isolates. This study confirms that CA-MRSA strains carried more virulence genes, especially staphylococcal enterotoxins genes (*seb/sec/sem ~ o*). SEs are a major cause of staphylococcal food poisoning, associated with severe disease. SEB may suppress the motility of human poly-morphonuclear neutrophils through the inhibition of exoprotein expression, and allow MRSA to invade and damage tissues [25]. Previous studies have confirmed that the *sea* and *seb* genes are the most abundant toxin genes in clinical *S. aureus* isolates from patients in China [26, 27]. Consistently, *sea* gene was the predominant SE gene detected in this study. No differences between the two MRSA groups were detected nor correlation to the clinical diagnosis. Of note, the *seb* and *sec* genes were significantly associated with skin and soft tissue infection (*P* < 0.001), and proved to be significant markers of CA-MRSA isolates. This study confirms that HA-MRSA clones predominantly carry the egc cluster(*sea*, *seg*, *sei*, *sem*, *sen and seo*), as reported in Europe and the USA [28]. Notablely, the *tsst*-1 gene was only found in the strains of skin and tissue infection patients, suggesting that these patients may have a greater potential for developing fatal toxin shock syndrome (TSS) [16]. Additionally, three SE genes (*sed, see* and *sej*) and two ET genes (*eta* and *etb*) were not found in this study.

An important finding in this study was the high detection rate of the *lukF-PV* and *lukS-PV* gene in the 62 MRSA isolates, while appeared no significantly different between CA-MRSA (47.8 %) and HA-MRSA (43.6 %) groups. PVL is a pore-forming toxin encoded by *lukF-PV* and *lukS-PV*, belonging to a family of similar bi-component leukocidin toxins produced by staphylococci, which is postulated widely as the primary virulence determinant driving the epidemic spread of the major CA-MRSA clone worldwide [29, 30]. Although PVL is known as a common virulence factor of CA-MRSA, several studies showed the HA-MRSA isolates had a considerable rate of PVL positivity in their regions [31]. Zetola et al. found that the prevalence of PVL tended to be elevated in nosocomial infections, indicating possible clonal expansion of those CA-MRSA into the hospital environment [32].

From the above, PVL may no longer be a reliable marker for CA-MRSA isolates, rather all MRSA may be important reservoir of PVL toxin. This indicates that HA-MRSA with typical molecular characteristics of CA-MRSA (SCC*mec* type IVa, V and PVL positive) have emerged as an important cause of healthcare-associated infections.

The current work has several limitations. Most importantly, the low number of studied strains and the lack of bacteremia cases limited the broad representative significance of the research. Secondly, the protein expression of virulence gene was not studied yet. It was reported that the expression of drug-resistance genes in *S. aureus* strains could decrease the expression of virulence factors [25, 33], which need further studied. Thirdly, spa typing was not performed in the current study, and this is something that should be conducted in future analyses. Despite the above limitations, our results add to the epidemiological information on CA-MRSA and HA-MRSA, could contribute to more effective disease control of MRSA infections.

## Conclusions

These data indicate a high prevalence of ST239-III-MRSA in both CA-MRSA and HA-MRSA isolates identified within 48 h after admission in Sun Yat-Sen Memorial Hospital, Guangzhou, but obviously different characteristic of molecular epidemiology and virulence genes profile between the CA and HA-MRSA group. Furthermore, this study indicates a possible transmission from HA-MRSA to CA-MRSA, that may be useful for understanding the overlap of the definition of the above two, and for designing strategies to prevent the MRSA dissemination.

### Abbreviations

AST, antimicrobial susceptibility test; CLSI, Clinical and Laboratory Standards Institute; CA-MRSA, community-acquired methicillin-resistant *Staphylococcus aureus*; ET, exfoliative toxin; HA-MRSA, hospital-acquired methicillin-resistant *Staphylococcus aureus*; *hla ~ hlg,* α-hemolysin ~ γ-hemolysin gene; MLST, multilocus sequence typing; MRSA, methicillin-resistant *Staphylococcus aureus*; PFGE, pulsed-field gel electrophoresis; PVL, Panton-Valentine leukocidin gene ; SE, staphylococcal enterotoxins; ST, sequence type; SCC*mec*, staphylococcal cassette chromosome mec; *sea ~ seo*, staphylococcal enterotoxins A ~ O gene; *tsst*-1, toxic shock syndrome toxin 1 gene.

## Additional files

Additional file 1: The statement on ethics approval. (JPG 256 kb) Additional file 2: Table S1. The antibiotic resistance of 62 methicillin-resistant *S. aureus* isolates. **Table S2.** The SCC*mec* type distribution of CA-MRSA and HA-MRSA. **Table S3.** MLST type distribution of CA-MRSA and HA-MRSA. **Table S4.** SCC*mec* type and MLST type distribution of 62 MRSA isolates. (PDF 202 kb) Additional file 3: Figure S1. Agarose gel image of virulence factor genes of partial positive strains in the 62 MRSA isolates. (A ~ M), Virulence factor genes detected by PCR. Marker refers to DNA marker (100 bp ladder); # numbers, isolate number; *Sea ~ seo*, staphylococcal enterotoxins A ~ O gene; *hla,* α-hemolysin gene; *hlb*, β-hemolysin gene; *hlg*, γ-hemolysin gene; *tsst*-1,toxic shock syndrome toxin 1 gene; PVL, Panton-Valentine leukocidin gene ; PCR, polymerase chain reaction. (PDF 280 kb) Additional file 4: Figure S2. Genetic relatedness among the 60 MRSA strains. Phylogenetic relationships were obtained by PFGE. Cluster analysis was performed with the software program BioNumerics 5.0 (Maths, Belgium) using the Dice coefficient and the unweighted pair group method(UPGMA). The isolates with the similarity over 75 % were clustered in patterns. PFGE, pulsed-field gel electrophoresis; CA/HA, community-acquired MRSA or hospital-acquired MRSA; PVL, Panton-Valentine leukocidin gene; SCC*mec*, staphylococcal cassette chromosome mec; MLST, multilocus sequence typing; +, positive. (PDF 24 kb)

## References

[1] Jevons MP, Coe AW, Parker MT (1963). Methicillin resistance in staphylococci. Lancet.

[2] Hiramatsu K, Okuma K, Ma XX, Yamamoto M, Hori S, Kapi M (2002). New trends in Staphylococcus aureus infections: glycopeptide resistance in hospital and methicillin resistance in the community. Curr Opin Infect Dis.

[3] Salgado CD, Farr BM, Calfee DP (2003). Community-acquired methicillin-resistant Staphylococcus aureus: a meta-analysis of prevalence and risk factors. Clin Infect Dis.

[4] Hsueh PR, Teng LJ, Chen WH, Pan HJ, Chen ML, Chang SC, Luh KT, Lin FY (2004). Increasing prevalence of methicillin-resistant Staphylococcus aureus causing nosocomial infections at a university hospital in Taiwan from 1986 to 2001. Antimicrob Agents Chemother.

[5] David MZ, Cadilla A, Boyle-Vavra S, Daum RS (2014). Replacement of HA-MRSA by CA-MRSA infections at an academic medical center in the midwestern United States, 2004–5 to 2008. PLoS One.

[6] Chuang YY, Huang YC (2013). Molecular epidemiology of community-associated meticillin-resistant Staphylococcus aureus in Asia. Lancet Infect Dis.

[7] Du J, Chen C, Ding B, Tu J, Qin Z, Parsons C, Salgado C, Cai Q, Song Y, Bao Q (2011). Molecular characterization and antimicrobial susceptibility of nasal Staphylococcus aureus isolates from a Chinese medical college campus. PLoS One.

[8] Geng W, Yang Y, Wu D, Zhang W, Wang C, Shang Y, Zheng Y, Deng L, Fu Z, Li X (2010). Community-acquired, methicillin-resistant Staphylococcus aureus isolated from children with community-onset pneumonia in China. Pediatr Pulmonol.

[9] Yao D, Yu FY, Qin ZQ, Chen C, He SS, Chen ZQ, Zhang XQ, Wang LX (2010). Molecular characterization of Staphylococcus aureus isolates causing skin and soft tissue infections (SSTIs). BMC Infect Dis.

[10] Scazzocchio F, Aquilanti L, Tabacchini C, Iebba V, Passariello C. Microbiological and molecular characterization of nosocomial and community Staphylococcus aureus isolates. Epidemiol Infect. 2011;139:613–622. 10.1017/S095026881000138X 20561388

[11] Portillo BC, Moreno JE, Yomayusa N, Alvarez CA, Cardozo BE, Perez JA, Diaz PL, Ibanez M, Mendez-Alvarez S, Leal AL, Gomez NV. Molecular epidemiology and characterization of virulence genes of community-acquired and hospital-acquired methicillin-resistant Staphylococcus aureus isolates in Colombia. Int J Infect Dis. 2013;17:e744-749. 10.1016/j.ijid.2013.02.029 23623704

[12] Gillet Y, Issartel B, Vanhems P, Fournet JC, Lina G, Bes M, Vandenesch F, Piemont Y, Brousse N, Floret D, Etienne J (2002). Association between Staphylococcus aureus strains carrying gene for Panton-Valentine leukocidin and highly lethal necrotising pneumonia in young immunocompetent patients. Lancet.

[13] Chen X, Yang HH, Huangfu YC, Wang WK, Liu Y, Ni YX, Han LZ (2012). Molecular epidemiologic analysis of Staphylococcus aureus isolated from four burn centers. Burns.

[14] Soriano A, Marco F, Martinez JA, Pisos E, Almela M, Dimova VP, Alamo D, Ortega M, Lopez J, Mensa J (2008). Influence of vancomycin minimum inhibitory concentration on the treatment of methicillin-resistant Staphylococcus aureus bacteremia. Clin Infect Dis.

[15] Diep BA, Chambers HF, Graber CJ, Szumowski JD, Miller LG, Han LL, Chen JH, Lin F, Lin J, Phan TH (2008). Emergence of multidrug-resistant, community-associated, methicillin-resistant Staphylococcus aureus clone USA300 in men who have sex with men. Ann Intern Med.

[16] Chen X, Wang WK, Han LZ, Liu Y, Zhang H, Tang J, Liu QZ, Huangfu YC, Ni YX (2013). Epidemiological and genetic diversity of Staphylococcus aureus causing bloodstream infection in Shanghai, 2009–2011. PLoS One.

[17] Wang CC, Lo WT, Chu ML, Siu LK (2004). Epidemiological typing of community-acquired methicillin-resistant Staphylococcus aureus isolates from children in Taiwan. Clin Infect Dis.

[18] Lee TM, Yang MC, Yang TF, Lee PL, Chien HI, Hsueh JC, Chang SH, Hsu CH, Chien ST (2015). Molecular Characterization of Community- and Healthcare-Associated Methicillin-Resistant Staphylococcus aureus Isolates in Southern Taiwan. Microb Drug Resist.

[19] Chen B, Dai X, He B, Pan K, Li H, Liu X, Bao Y, Lao W, Wu X, Yao Y, Huang S (2015). Differences in Staphylococcus aureus nasal carriage and molecular characteristics among community residents and healthcare workers at Sun Yat-Sen University, Guangzhou, Southern China. BMC Infect Dis.

[20] Popovich K, Hota B, Rice T, Aroutcheva A, Weinstein RA (2007). Phenotypic prediction rule for community-associated methicillin-resistant Staphylococcus aureus. J Clin Microbiol.

[21] Otter JA, French GL (2011). Utility of antimicrobial susceptibility-based algorithms for the presumptive identification of genotypically-defined community-associated methicillin-resistant Staphylococcus aureus at a London teaching hospital. Eur J Clin Microbiol Infect Dis.

[22] Gbaguidi-Haore H, Thouverez M, Couetdic G, Cholley P, Talon D, Bertrand X (2009). Usefulness of antimicrobial resistance pattern for detecting PVL- or TSST-1-producing meticillin-resistant Staphylococcus aureus in a French university hospital. J Med Microbiol.

[23] Liu Q, Han L, Li B, Sun J, Ni Y (2012). Virulence characteristic and MLST-agr genetic background of high-level mupirocin-resistant, MRSA isolates from Shanghai and Wenzhou. China PLoS One.

[24] Yu F, Liu Y, Lv J, Qi X, Lu C, Ding Y, Li D, Liu H, Wang L (2015). Antimicrobial susceptibility, virulence determinant carriage and molecular characteristics of Staphylococcus aureus isolates associated with skin and soft tissue infections. Braz J Infect Dis.

[25] Yu F, Li T, Huang X, Xie J, Xu Y, Tu J, Qin Z, Parsons C, Wang J, Hu L, Wang L (2012). Virulence gene profiling and molecular characterization of hospital-acquired Staphylococcus aureus isolates associated with bloodstream infection. Diagn Microbiol Infect Dis.

[26] Wu D, Li X, Yang Y, Zheng Y, Wang C, Deng L, Liu L, Li C, Shang Y, Zhao C (2011). Superantigen gene profiles and presence of exfoliative toxin genes in community-acquired meticillin-resistant Staphylococcus aureus isolated from Chinese children. J Med Microbiol.

[27] He W, Chen H, Zhao C, Zhang F, Li H, Wang Q, Wang X, Wang H (2013). Population structure and characterisation of Staphylococcus aureus from bacteraemia at multiple hospitals in China: association between antimicrobial resistance, toxin genes and genotypes. Int J Antimicrob Agents.

[28] Diep BA, Carleton HA, Chang RF, Sensabaugh GF, Perdreau-Remington F (2006). Roles of 34 virulence genes in the evolution of hospital- and community-associated strains of methicillin-resistant Staphylococcus aureus. J Infect Dis.

[29] Tristan A, Ferry T, Durand G, Dauwalder O, Bes M, Lina G, Vandenesch F, Etienne J (2007). Virulence determinants in community and hospital meticillin-resistant Staphylococcus aureus. J Hosp Infect.

[30] Maree CL, Daum RS, Boyle-Vavra S, Matayoshi K, Miller LG (2007). Community-associated methicillin-resistant Staphylococcus aureus isolates causing healthcare-associated infections. Emerg Infect Dis.

[31] Li DZ, Chen YS, Yang JP, Zhang W, Hu CP, Li JS, Mu L, Hu YH, Geng R, Hu K (2011). Preliminary molecular epidemiology of the Staphylococcus aureus in lower respiratory tract infections: a multicenter study in China. Chin Med J (Engl).

[32] Zetola N, Francis JS, Nuermberger EL, Bishai WR (2005). Community-acquired meticillin-resistant Staphylococcus aureus: an emerging threat. Lancet Infect Dis.

[33] Collins J, Rudkin J, Recker M, Pozzi C, O’Gara JP, Massey RC (2010). Offsetting virulence and antibiotic resistance costs by MRSA. Isme J.

