# Supplementary figures and images for: Molecular epidemiology and characteristic of virulence gene of community-acquired and hospital-acquired methicillin-resistant Staphylococcus aureus isolates in Sun Yat-sen Memorial hospital, Guangzhou, Southern China
Source: BMC Infect Dis. 2016 Jul 22;16:339. doi: 10.1186/s12879-016-1684-y (PMC4957337; doi:10.1186/s12879-016-1684-y)

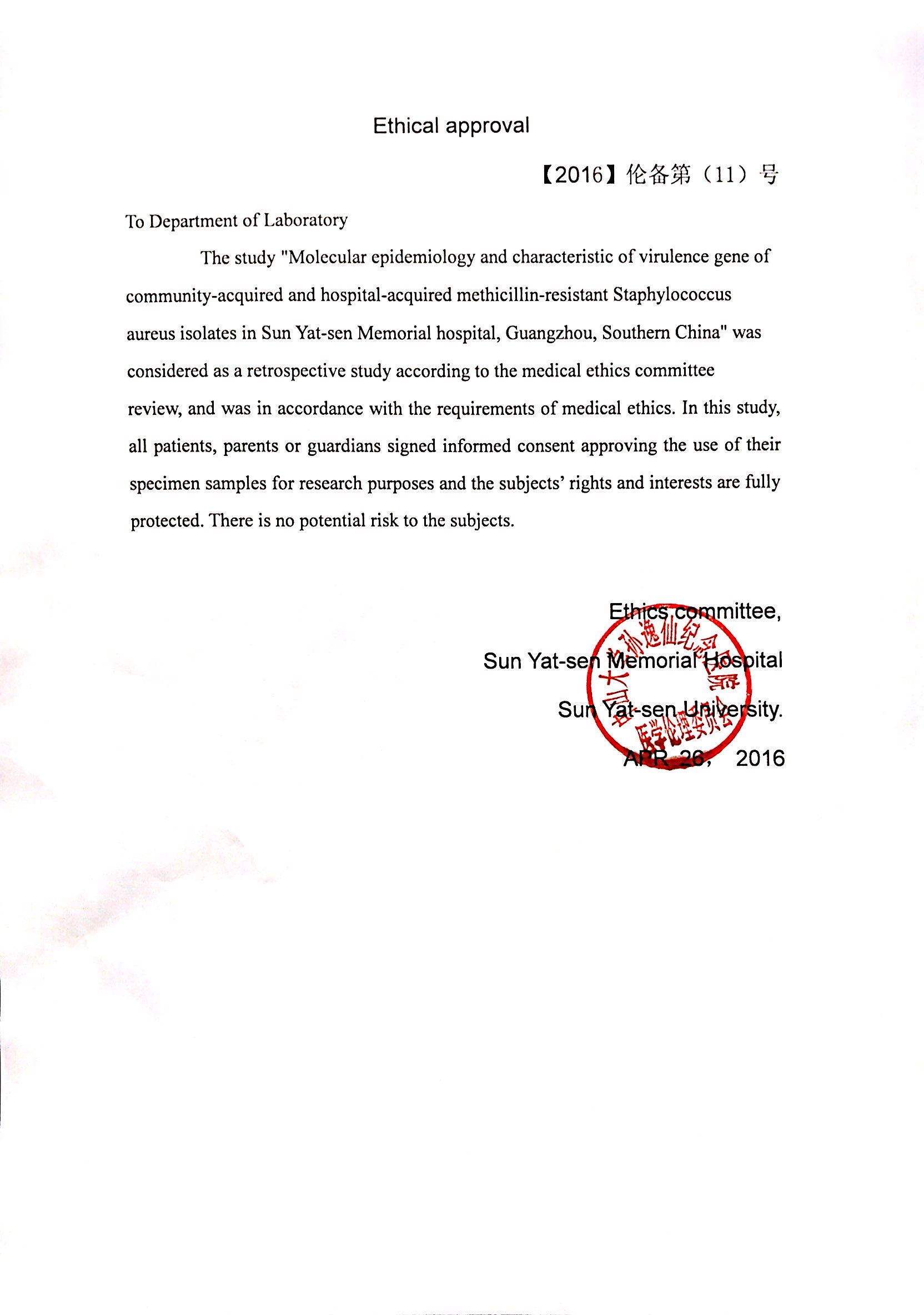

Supplement: Additional file 1: — The statement on ethics approval. (JPG 256 kb) [file 12879_2016_1684_MOESM1_ESM.jpg]

**A** *sea*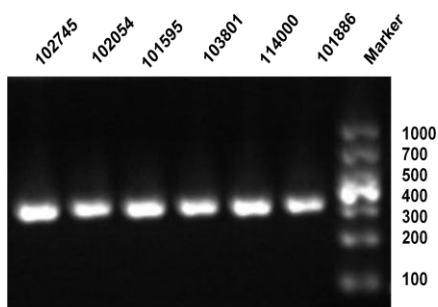**B** *seb*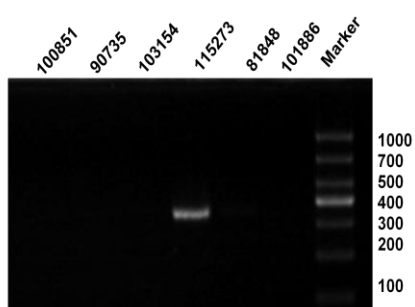**C** *sec*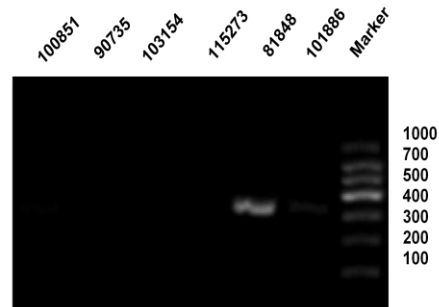**D** *seg*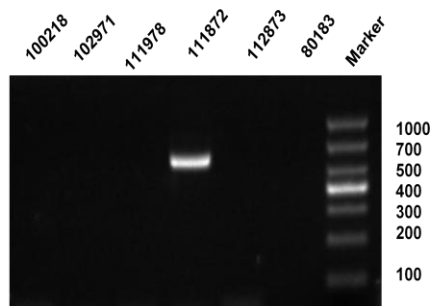**E** *sei*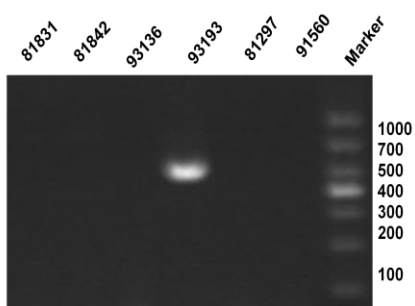**F** *sen*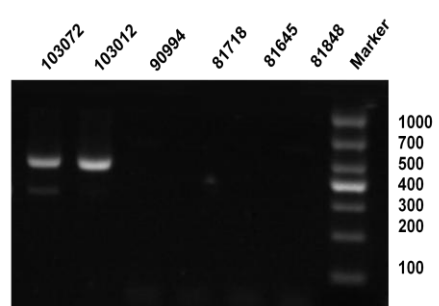**G** *sem*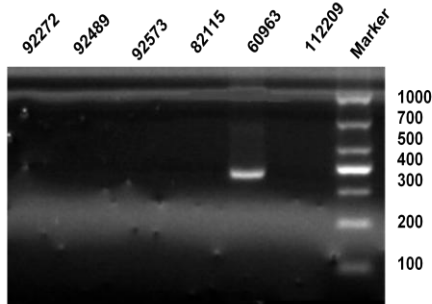**H** *seo*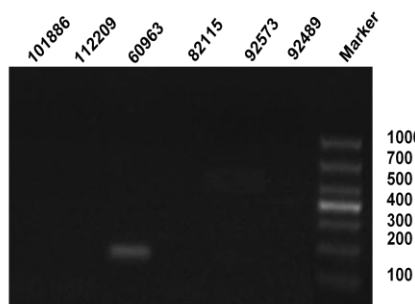**I** *hla*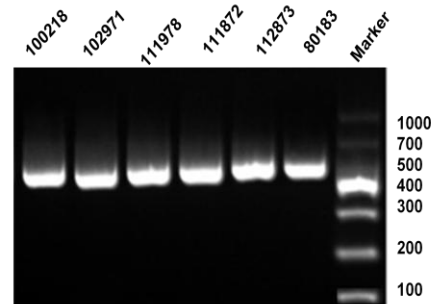**J** *h1b*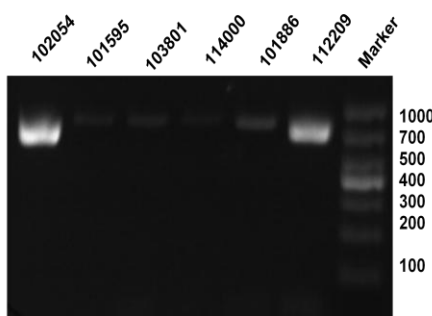**K** *h1g*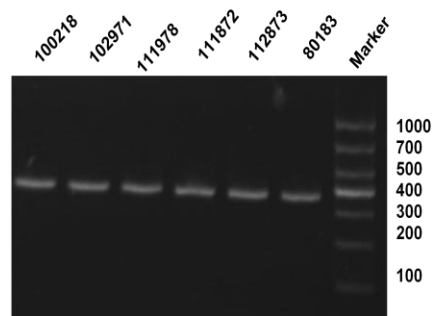**L** *tsst-1*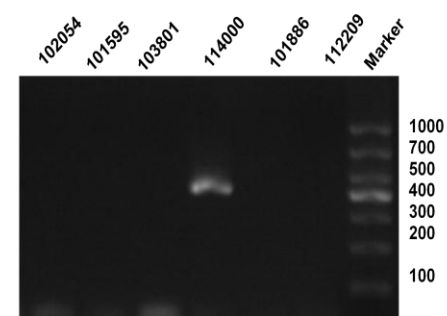**M** *pvl*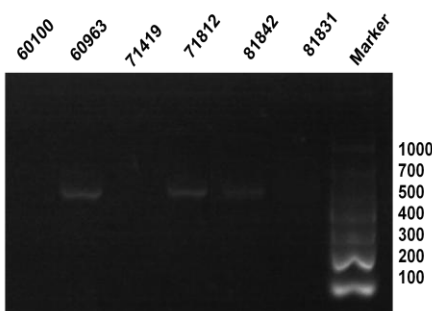

Supplement: Additional file 3: Figure S1. — Agarose gel image of virulence factor genes of partial positive strains in the 62 MRSA isolates. (A ~ M), Virulence factor genes detected by PCR. Marker refers to DNA marker (100 bp ladder); # numbers, isolate number; Sea ~ seo, staphylococcal enterotoxins A ~ O gene; hla, α-hemolysin gene; hlb, β-hemolysin gene; hlg, γ-hemolysin gene; tsst-1,toxic shock syndrome toxin 1 gene; PVL, Panton-Valentine leukocidin gene ; PCR, polymerase chain reaction. (PDF 280 kb) [file 12879_2016_1684_MOESM3_ESM.pdf]

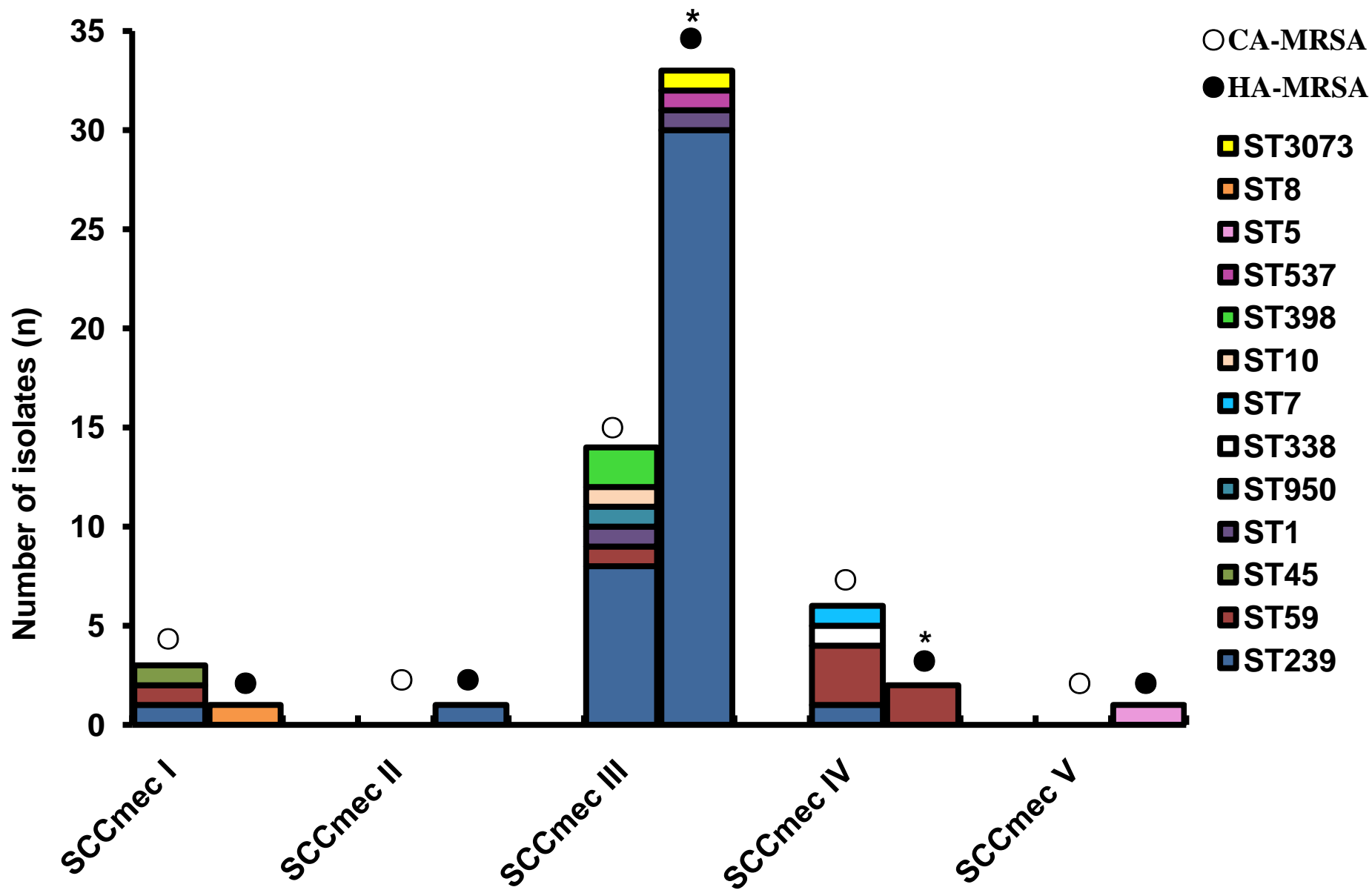

Supplement: Additional file 4: Figure S2. — Genetic relatedness among the 60 MRSA strains. Phylogenetic relationships were obtained by PFGE. Cluster analysis was performed with the software program BioNumerics 5.0 (Maths, Belgium) using the Dice coefficient and the unweighted pair group method(UPGMA). The isolates with the similarity over 75 % were clustered in patterns. PFGE, pulsed-field gel electrophoresis; CA/HA, community-acquired MRSA or hospital-acquired MRSA; PVL, Panton-Valentine leukocidin gene; SCCmec, staphylococcal cassette chromosome mec; MLST, multilocus sequence typing; +, positive. (PDF 24 kb) [file 12879_2016_1684_MOESM4_ESM.pdf]
