# Supplementary material for: Molecular epidemiology and characteristic of virulence gene of community-acquired and hospital-acquired methicillin-resistant Staphylococcus aureus isolates in Sun Yat-sen Memorial hospital, Guangzhou, Southern China
Source: BMC Infect Dis. 2016 Jul 22;16:339. doi: 10.1186/s12879-016-1684-y (PMC4957337; doi:10.1186/s12879-016-1684-y)
Supplement: Additional file 2: Table S1. — The antibiotic resistance of 62 methicillin-resistant S. aureus isolates. Table S2. The SCCmec type distribution of CA-MRSA and HA-MRSA. Table S3. MLST type distribution of CA-MRSA and HA-MRSA. Table S4. SCCmec type and MLST type distribution of 62 MRSA isolates. (PDF 202 kb) [file 12879_2016_1684_MOESM2_ESM.pdf]

Table S1 The antibiotic resistance of 62 methicillin-resistant *S. aureus* isolates.

antibiotic	Resistance rate (%)		Overall (n=62)	*P*-value
	CA-MRSA (n=23)	HA-MRSA （n=39）		
CIP	34.8	89.7	69.4	<0.001
MFX	17.4	39.5	31.1	0.001
E	95.7	91.9	93.3	0.373
DA	95.7	92.3	93.5	0.373
RD	4.3	33.3	22.6	<0.001
CN	21.7	84.6	61.3	<0.001
SXT	34.8	41.0	38.7	0.466
TET	65.2	79.4	74.2	0.040
VA	0	0	0	
TEC	0	0	0	
LZD	0	0	0	

CIP, ciprofloxacin; MFX, moxifloxacin; E, erythromycin; DA, clindamycin; RD, rifampicin;CN, gentamicin; SXT, trimethoprim/sulfamethoxazole; TET, tetracycline; VA, vancomycin; TEC, teicoplanin; LZD, linezolid; CA-MRSA, community-acquired methicillin-resistant *Staphylococcus aureus*; HA-MRSA, hospital-acquired methicillin-resistant *Staphylococcus aureus*.

Table S2 The SCC*mec* type distribution of CA-MRSA and HA-MRSA (n, %).

SCC*mec* type	Total (n=62)	CA-MRSA (n=23)	HA-MRSA (n=39)	*P*-value
SCC*mec* I	4(6.5)	3(13.0)	1(2.6)	0.061
SCC*mec* II	1(1.6)	-	1(2.6)	
SCC*mec* III	48(77.4)	14(60.9)	34(87.2)	<0.001
SCC*mec* IV(a and d)	8(12.9)	6(26.0)	2(5.1)	<0.001
SCC*mec* V	1(1.6)	-	1(2.6)	
Total	62(100)	23(100)	39(100)	

SCC*mec*, staphylococcal cassette chromosome mec; -, not detected; CA-MRSA, community-acquired methicillin-resistant *Staphylococcus aureus*; HA-MRSA, hospital-acquired methicillin-resistant *Staphylococcus aureus*.

Table S3 MLST type distribution of CA-MRSA and HA-MRSA (n, %).

ST	Total (n=62)	CA-MRSA (n=23)	HA-MRSA (n=39)	*P*-value
ST239	41(66.1)	10(43.4)	31(79.5)	<0.001
ST59	7(11.3)	5(21.7)	2(5.1)	0.003
ST1	2(3.2)	1(4.3)	1(2.6)	1.000
ST45	1(1.6)	1(4.3)	-	
ST950	1(1.6)	1(4.3)	-	
ST338	1(1.6)	1(4.3)	-	
ST7	1(1.6)	1(4.3)	-	
ST10	1(1.6)	1(4.3)	-	
ST398	2(3.2)	2(8.6)	-	
ST537	1(1.6)	-	1(2.6)	
ST5	1(1.6)	-	1(2.6)	
ST8	1(1.6)	-	1(2.6)	
ST3703	1(1.6)	-	1(2.6)	
Non type	1(1.6)	-	1(2.6)	

MLST, multilocus sequence typing; ST, sequence type; -, not detected; CA-MRSA, community-acquired methicillin-resistant *Staphylococcus aureus*; HA-MRSA, hospital-acquired methicillin-resistant *Staphylococcus aureus*.

Table S4 SCC*mec* type and MLST type distribution of 62 MRSA isolates.

ST	SCC*mec* I （n=4）	SCC*mec* II (n=1)	SCC*mec* III (n=46)	SCC*mec* IVa (n=5)	SCC*mec* IVd （n=3）	SCC*mec* V （n=1）
ST239	1(CA)	1(HA)	8(CA), 30 (HA)	-	1(CA)	-
ST59	1(CA)	-	1(CA)	2 (HA), 2(CA)	1(CA)	-
ST45	1(CA)	-	-	-	-	-
ST1	-	-	1 (CA), 1(HA)	-	-	-
ST950	-	-	1(CA)	-	-	-
ST338	-	-	-	-	1(CA)	-
ST7	-	-	-	1(CA)	-	-
ST10	-	-	1(CA)	-	-	-
ST398	-	-	2(CA)	-	-	-
ST537	-	-	1(HA)	-	-	-
ST5	-	-	-	-	-	1(HA)
ST8	1(HA)	-	-	-	-	-
New type	-	-	2(HA)	-	-	-

SCC*mec*, staphylococcal cassette chromosome mec; MLST, multilocus sequence typing; ST, sequence type; -, not detected.
